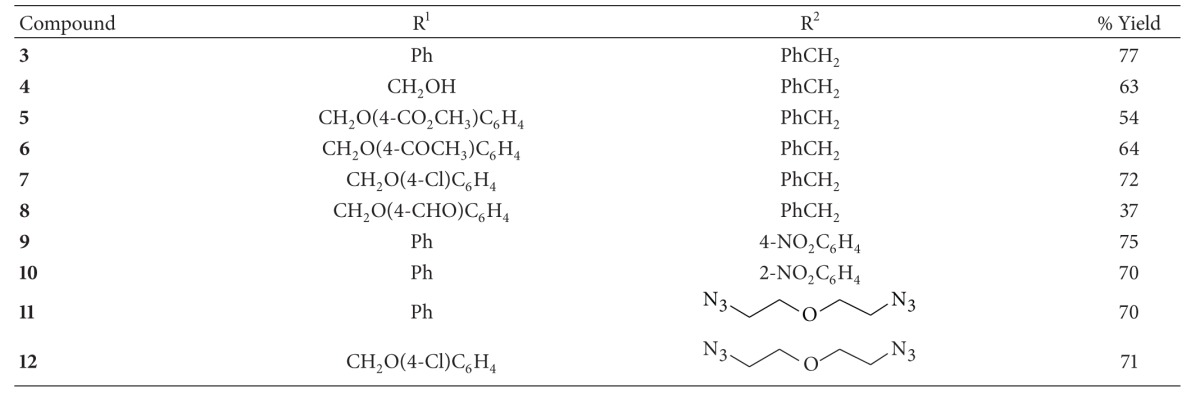# Alkyne-Azide Cycloaddition Catalyzed by Silver Chloride and “Abnormal” Silver *N*-Heterocyclic Carbene Complex

**DOI:** 10.1155/2013/186537

**Published:** 2013-11-06

**Authors:** Aldo I. Ortega-Arizmendi, Eugenia Aldeco-Pérez, Erick Cuevas-Yañez

**Affiliations:** ^1^Centro Conjunto de Investigación en Química Sustentable UAEM-UNAM, Carretera Toluca-Atlacomulco Km. 14.5, 50200 Toluca, MEX, Mexico; ^2^Facultad de Química, Universidad Autónoma de Querétaro, Centro Universitario Cerro de las Campanas s/n, 76010 Querétaro, QRO, Mexico

## Abstract

A library of 1,2,3-triazoles was synthesized from diverse alkynes and azides using catalytic amounts of silver chloride instead of copper compounds. In addition, a novel “abnormal” silver *N*-heterocyclic carbene complex was tested as catalyst in this process. The results suggest that the reaction requires only 0.5% of silver complex, affording 1,2,3-triazoles in good yields.

## 1. Introduction

The study about the comprehension and applications of the classical (3 + 2) dipolar cycloaddition, the Huisgen Reaction, was revolutionized with the introduction of copper catalysts to this process, which increases selectivity and efficiency to the reaction, allowing diverse applications in several solvents and conditions for an important number of purposes. These characteristics make the copper catalyzed alkyne azide cycloaddition (CuAAC) in the most important “Click” reaction [1]. 

An essential component in this reaction is the catalyst, based mainly on a copper (I) salt. In this regard, a wide variety of copper compounds and conditions have been proposed and developed in order to optimize this process [2, 3]. In addition, some research groups have explored other metals as alternative catalyst sources, such as ruthenium [4–6], magnesium (through Grignard reagents) [7], and zinc compounds [8]. In spite of these examples, the number of reports about alkyne azide cycloadditions catalyzed by different metals is still limited. 

Recently, McNulty and coworkers demonstrated that P,O type silver complexes can catalyze AAC without other copper additives [9, 10]. These promising reports carried us to report our recent results in this area.

## 2. Experimental Section

The starting materials were purchased from Aldrich Chemical Co. and were used without further purification. Solvents were distilled before use. Silica plates of 0.20 mm thickness were used for thin layer chromatography. Melting points were determined with a Fisher-Johns melting point apparatus, and they are uncorrected. ^1^H and ^13^C NMR spectra were recorded using a Varian 500; the chemical shifts (*δ*) are given in ppm relative to TMS as internal standard (0.00). For analytical purposes, the mass spectra were recorded on a Shimadzu GCMS-QP2010 Plus in the EI mode, 70 eV, 200°C via direct inlet probe. Only the molecular and parent ions (*m/z*) are reported. IR spectra were recorded on a Bruker Tensor 27 equipment. 

1,3-Bis(2,6-diisopropylphenyl)-2,4-diphenyl-imidazolium chloride (**13**) was synthesized according to a previous report [11].

For the RX diffraction studies, crystals of compound **14** were obtained by slow evaporation of a dilute EtOH solution, and the reflections were acquired with a Bruker diffractometer. Three standard reflections every 97 reflections were used to monitor the crystal stability. The structure was solved by direct methods; missing atoms were found by difference-Fourier synthesis and refined on F2 by a full-matrix least-squares procedure using anisotropic displacement parameters using SHELX-97. Crystallographic data for the structure reported in this paper have been deposited with the Cambridge Crystallographic Data Centre (CCDC No. 900045). Copies of available materials can be obtained free of charge on application to the Director, CCDC, 12 Union Road, Cambridge CB2 IEZ, UK (facsimile: (44) 01223 336033); e-mail: deposit@ccdc.ac.uk.

### 2.1. Synthesis of Silver Complex of 1,3-Bis(2,6-diisopropylphenyl)-2,4-diphenyl-imidazolium Chloride

 1,3-Bis(2,6-diisopropylphenyl)-2,4-diphenyl-imidazolium chloride (1 g, 1.6 mmoL) were dissolved in dry THF (20 mL) and cooled down to −70°C. After 15 min, a 1 M solution of NaHMDS in THF (4 mL) was added, and the resulting mixture was stirred 1 hour at room temperature. After precipitation of all solids contained in the reaction mixture, the solution was filtered, and a suspension of silver chloride (229 mg, 1.6 mmoL) in THF (30 mL) was added and the reaction mixture was stirred 12 hours at room temperature. After evaporation of the solvent under vacuum, purification was done by adding 30 mL of dichloromethane, filtering from a black precipitate, and pumping down the solvent under reduced pressure. 586 mg (54% yield) of a gray powder were obtained. ^1^H NMR: (300 MHz, CDCl_3_ + DMSO-d_6_) *δ* 0.81 (d, *J *= 6.9 Hz, 3H), 0.82 (d,   *J* = 6.9 Hz, 3H), 0.95 (d, *J* = 6.9 Hz, 3H), 1.38 (d,  *J* = 6.6 Hz, 3H), 2.57 (sept, *J* = 6.9 Hz, 1H), 2.65 (sept., *J* = 6.9 Hz, 1H), 6.88–6.85 (m, 2H), 7.09–7.04 (m, 2H), 7.33–7.18 (m, 8H), and 7.48–7.38 (m, 4H). ^13^C NMR: (75 MHz, CDCl_3_ + DMSO-d_6_) *δ* 22.4, 23.2, 25.5, 25.6, 28.4, 28.5, 124.6, 125.1, 128.0, 128.1, 128.9, 129.2, 130.2, 130.4, 131.1, 135.8, 141.9, 142.0, 144.4, 144.6, and 161.4 (dd, *J* C-107Ag = 247 Hz, *J *C-109Ag = 265 Hz, C-Ag). Crystal and structure parameters of silver complex C_40_H_46_AgCl_3_N_2_: volume = 1863.51(11) Å3, triclinic, space group *P* − 1, *a* = 9.8904(3) Å, *b* = 11.3602(4) Å, *c* = 17.9355(6) Å, *α* = 95.1220(10)°, *β* = 92.6030 (10)°, *γ* = 111.2940(10)°, *ρ*calcd = 1.370 g/cm^3^, Mo-radiation (*λ* = 0.71073 Å), *T* = 100(2) K, reflections collected = 19486, independent reflections = 6602 (Rint. = 0.0316), absorption coefficient *μ* = 0.786 mm^−1^, 433 variables converged at R1 = 2.96%, wR2 = 7.70% for all data, with intensity *I* > 2*σ*(I), the final difference map was 0.513 and −0.437 eÅ^−3^.

### 2.2. Synthesis of 1,2,3-Triazoles Performed under AgCl Catalysis

*General Procedure*. AgCl (0.005 g, 0.035 mmol) was added to a stirred solution containing the corresponding alkyne (1.0 mmol) and the appropriate azide (1.1 mmol) in H_2_O (3 mL) and acetone (1 mL). The resulting reaction mixture was stirred at room temperature for 24 h. The acetone was removed *in vacuo*, and CH_2_Cl_2_ (20 mL) was added. The organic layer was separated, and the aqueous phase was extracted with CH_2_Cl_2_  (3 × 10 mL). The organic phases were joined, dried over Na_2_SO_4_ and filtered. The solvent was removed under reduced pressure and the final product was purified by crystallization.

### 2.3. Synthesis of 1,2,3-Triazoles Performed under Silver Complex Catalysis

*General Procedure*. The silver complex **14** (0.0005 mmol) was added to a stirred solution containing the corresponding alkyne (1.0 mmol) and the appropriate azide (1.05 mmol) in THF (4 mL). The resulting reaction mixture was stirred at room temperature for 24 h. The acetone was removed *in vacuo*, and CH_2_Cl_2_ (20 mL) was added. The organic layer was separated, and the aqueous phase was extracted with CH_2_Cl_2_  (3 × 10 mL). The organic phases were joined, dried over Na_2_SO_4_, and filtered. The solvent was removed under reduced pressure, and the final product was purified by crystallization.

### 2.4.  1-Benzyl-4-phenyl-1,2,3-triazole (**3**)

 Phenylacetylene and benzyl azide afforded 1-benzyl-4-phenyl-1,2,3-triazole as white solid. Yields: 150.8 mg (64%, under AgCl catalysis) and 181.1 mg (77%, under silver complex catalysis). m.p. 132°C (lit. 130-130.9°C) [12]. IR (ATR) *ν*_max⁡_/cm^−1^: 3250, 2850, 1650, and 1600. ^1^H NMR (500 MHz, CDCl_3_) *δ*/ppm: 5.59 (s, 2H), 7.33–7.39 (m, 1H), 7.41-7.41 (m, 4H), 7.68–7.82 (m, 2H), and 7.68 (s, 1H). ^13^C NMR (125 MHz, CDCl_3_) *δ*/ppm: 54.2 (CH_2_), 119.5 (CH), 125.6 (2 × CH), 127.9 (2 × CH), 128.1 (CH), 128.7 (CH), 128.8 (2 × CH), 129.1 (2 × CH), 130.5(CH), 134.6 (C), and 148.2 (C). MS (EI^+^) *m/z* (%): 235[M]^+^ (21), 206 [M–HN_2_]^+^ (74), 116 [M–C_6_H_5_N_3_]^+^ (100), and 91 [C_6_H_5_CH_2_]^+^ (94).

### 2.5. (1-Benzyl-1,2,3-triazol-4-yl)-methanol (**4**)

 Propargyl alcohol and benzyl azide afforded (1-benzyl-1,2,3-triazol-4-yl)-methanol as white solid. Yields: 109.7 mg (58%, under AgCl catalysis) and 120.3 mg (63%, under silver complex catalysis). m.p. 77.8°C (lit. 76-77°C) [13]. IR (ATR) *ν*_max⁡_/cm^−1^: 3330, 2850, and 1600. ^1^H NMR (500 MHz, CDCl_3_) *δ*/ppm: 3.36 (s, 1H), 4.51 (s, 2H), 5.57 (s, 2H), 7.30–7.37 (m, 5H), and 8.00 (s, 1H). ^13^C NMR (125 MHz, CDCl_3_) *δ*/ppm: 53.1 (CH_2_), 55.4 (CH_2_), 123.2 (CH), 128.3 (2 × CH), 128.5 (CH), 129.1 (2 × CH), 136.6 (C), and 148.7 (C). MS (EI^+^) *m/z *(%): 189 [M]^+^ (4), 91 [C_6_H_5_CH_2_] + (100).

### 2.6.  4-(1-Benzyl-1,2,3-triazol-4-ylmethoxy)-benzoic Acid Methyl Ester (**5**)

4-Prop-2-ynyloxy-benzoic acid methyl ester and benzyl azide afforded 4-(1-benzyl-1,2,3-triazol-4-ylmethoxy)-benzoic acid methyl ester as white solid. Yields: 231.6 mg (72%, under AgCl catalysis) and 174.3 mg (54%, under silver complex catalysis). m.p. 145.7°C. IR (ATR) *ν*_max⁡_/cm^−1^: 1753, 1650, 1600. ^1^H NMR (500 MHz, CDCl_3_) *δ*/ppm: 3.87 (s, 3H), 5.22 (s, 2H), 5.53 (s, 2H), 6.96–6.99 (dd, 2H, *J* = 3 Hz), 7.96–7.99 (dd, 2H, *J* = 3 Hz), 7.26–7.28 (m, 2H), 7.35–7.39 (m, 3H), 7.53 (s, 1H). ^13^C NMR (125 MHz, CDCl_3_) *δ*/ppm: 51.8 (CH_3_), 54.3 (CH_2_), 62.1 (CH_2_), 114.2 (2 × CH), 122.7 (C), 123.1 (CH), 127.6 (2 × CH), 128.8 (CH), 129.4 (2 × CH), 131.6 (2 × CH), 134.0 (C), 143.9 (C), 161.8 (C), 166.7 (C). MS (EI+) *m/z* (%): 323 [M]^+^ (15), 91 [C_6_H_5_CH_2_]^+^ (100), 144 [C_10_H_10_N]^+^ (95), 172 [C_10_H_10_N_3_]^+^ (45). HRMS (EI): calcd. for C_18_H_17_N_3_O_3_: 323.1270; found: 323.1274.

### 2.7.  1-Benzyl-4-(4-methoxy-phenoxymethyl)-1,2,3-triazole (**6**)

1-Methoxy-4-prop-2-ynyloxy-benzene and benzyl azide afforded 1-benzyl-4-(4-methoxy-phenoxymethyl)-1,2,3-triazole as white solid. Yields: 162.3 mg (55%, under AgCl catalysis) and 189.3 mg (64%, under silver complex catalysis). m.p. 92.7°C (lit. 92-93°C) [14]. IR (ATR) *ν*_max⁡_/cm^−1^: 2950, 1650, and 1600. ^1^H NMR (500 MHz, CDCl_3_) *δ*/ppm: 3.76 (s, 3H), 5.13 (s, 2H), 5.52 (s, 2H), 6.80–6.82 (dd, 2H, *J* = 2 Hz, *J* = 9 Hz), 6.88–6.90 (dd, 2H, *J* = 2 Hz, *J* = 9 Hz), 7.26-7.27 (m, 2H), 7.36-7.38 (m, 3H), and 7.50 (s, 1H). ^13^C NMR (125 MHz, CDCl_3_) *δ*/ppm: 54.2 (CH_3_), 55.6 (CH_2_), 62.8 (CH_2_), 114.6 (2 × CH), 115.9 (2 × CH), 122.9 (CH), 128.1 (2 × CH), 128.8 (CH), 129.1 (2 × CH), 134.5 (C), 144.9 (C), 152.4 (C), and 154.3 (C). MS (EI^+^) *m/z* (%): 295 [M]^+^ (40), 91 [C_6_H_5_CH_2_]^+^ (100), 144 [C_10_H_10_N]^+^ (80), 124 [C_7_H_8_O_2_]^+^ (68), and 172 [C1_0_H_10_N_3_]^+^ (5).

### 2.8.  1-Benzyl-4-(4′-chlorophenoxymethyl)-1,2,3-triazole (**7**)

1-Chloro-4-prop-2-ynyloxy-benzene and benzyl azide afforded 1-benzyl-4-(4′-chlorophenoxymethyl)-1,2,3-triazole as white solid. Yields: 201.6 mg (67%, under AgCl catalysis) and 216.4 mg (72%, under silver complex catalysis). 102.3°C (lit. 102-103°C) [15]. IR (ATR) *ν*_max⁡_/cm^−1^: 1650, and 1600. ^1^H NMR (500 MHz, CDCl_3_) *δ*/ppm: 5.16 (s, 2H), 5.54 (s, 2H), 6.89–6.92 (dd, 2H,  *J* = 2 Hz,  *J* = 9 Hz), 7.22–7.25 (dd, 2H,  *J* = 3 Hz,  *J* = 9 Hz), 7.27–7.30 (m, 2H), 7.38–7.40 (m, 3H), and 7.53 (s, 1H). ^13^C NMR (125 MHz, CDCl_3_) *δ*/ppm: 54.2 (CH_2_), 62.2 (CH_2_), 116.0 (2 × CH), 122.6 (CH), 126.1 (C), 128.0 (2 × CH), 128.8, 129.4 (2 × CH) 129.7 (2 × CH), 134.3 (C), 144.1 (C), and 156.7 (C). MS (EI^+^) *m/z* (%): 299[M]^+^ (15), 91 [C_6_H_5_CH_2_]^+^ (100), 144 [C_10_H_10_N]^+^ (78), and 172 [C_10_H_10_N]^+^ (25).

### 2.9.  4-(1-Benzyl-1,2,3-triazol-4-ylmethoxy)-benzaldehyde (**8**)

4-Prop-2-ynyloxy-benzaldehyde and benzyl azide afforded 4-(1-benzyl-1,2,3-triazol-4-ylmethoxy)-benzaldehyde as white solid. Yields: 83.0 mg (28%, under AgCl catalysis) and 107.8 mg (37%, under silver complex catalysis). m.p. 79.3°C (lit. 79-80°C) [16]. IR (ATR) *ν*_max⁡_/cm^−1^: 1660, 1600. ^1^H NMR (500 MHz, CDCl_3_) *δ*/ppm: 5.26 (s, 2H), 5.54 (s, 2H), 7.07–7.09 (dd, 2H, *J* = 3 Hz, *J* = 9 Hz), 7.82-7.83 (dd, 2H, *J* = 3 Hz, *J* = 9 Hz), 7.26–7.29 (m, 2H), 7.36–7.38 (m, 3H), 7.54 (s, 1H), and 9.88 (s, 1H). ^13^C NMR (125 MHz, CDCl_3_) *δ*/ppm: 54.3 (CH_2_), 62.2 (CH_2_), 115.3 (2 × CH), 123.1 (CH), 128.2 (2 × CH), 128.9 (CH), 129.2 (2 × CH), 130.3 (C), 130.9 (2 × CH), 134.3 (C), 143.6 (C), 163.3 (C), and 190.7 (C). MS (EI^+^) *m/z* (%): 293[M]^+^ (5), 91 [C_6_H_5_CH_2_]^+^ (100), 144 [C_10_H_10_N]^+^ (65), and 172 [C_10_H_10_N_3_]^+^ (35).

### 2.10.  1-(4-Nitrophenyl)-4-phenyl-1,2,3-triazole (**9**)

Phenylacetylene and 1-azido-4-nitro-benzene afforded 1-(4-Nitrophenyl)-4-phenyl-1,2,3-triazole as orange solid. Yields: 204.7 mg (77%, under AgCl catalysis) and 200.7 mg (75%, under silver complex catalysis). m.p. 254°C (dec.) [17]. IR (ATR) *ν*_max⁡_/cm^−1^: 3050, 1600, 1550, and 1350. ^1^H NMR (500 MHz, DMSO-d_6_) *δ*/ppm: 7.40 (t, *J* = 7.5 Hz, 1H), 7.51 (t, *J* = 8.0 Hz, 2H), 7.95 (m, 2H), 8.25 (m, 2H), 8.48 (m, 2H), 9.51 (s, 1H). ^13^C NMR (125 MHz, DMSO-d_6_) *δ*/ppm: 120.4 (CH), 120.9 (2 × CH), 125.9 (2 × CH), 126.1 (2 × CH), 129.0 (CH), 129.5 (2 × CH), 130.2 (C), 141.3 (C), 147.1 (C), and 148.3 (C). MS (EI^+^) *m/z* (%): 266 [M]^+^ (15), 138 (75), 88 (100), and 57 (90).

### 2.11.  1-(2-Nitro-phenyl)-4-phenyl-1,2,3-triazole (**10**)

Phenylacetylene and 1-azido-2-nitro-benzene afforded 1-(2-nitrophenyl)-4-phenyl-1,2,3-triazole as orange solid. Yields: 190.8 mg (72%, under AgCl catalysis) and 186.0 mg (70%, under silver complex catalysis). m.p. 142.7°C (lit. 144-145°C) [18]. IR (ATR) *ν*_max⁡_/cm^−1^: 3050, 1600, 1550, 1350. ^1^H NMR (500 MHz, DMSO-d_6_) *δ*/ppm: 7.38 (m, *J* = 7.5 Hz, 1H), 7.47-7.48 (m*, J* = 7.5 Hz, 1H), 7.68–7.73 (m, 2H), 7.80-7.81 (m, 1H), 7.89–7.91 (d, *J* = 9 Hz, 2H), and 8.06–8.11 (m, 2H). ^13^C NMR (125 MHz, DMSO-d_6_) *δ*/ppm: 120.9 (CH), 125.6 (CH), 126.2 (CH), 127.8 (2 × CH), 128.8 (CH), 129.1 (2 × CH), 129.8 (C), 130.3 (C), 130.8 (CH), 133.8 (CH), 144.3 (C), and 148.4 (C). MS (EI^+^) *m/z* (%): 266 [M]^+^ (5), and 88 (100).

### 2.12.  4-Phenyl-1-{2-[2-(4-phenyl-1,2,3-triazol-1-yl)-ethoxy]-ethyl}-1,2,3-triazole

Phenylacetylene and 1-azido-2-(2-azido-ethoxy)-ethane afforded 4-phenyl-1-{2-[2-(4-phenyl-1,2,3-triazol-1-yl)-ethoxy]-ethyl}-1,2,3-triazole as white solid. Yields: 178.8 mg (62%, under AgCl catalysis) and 202.2 mg (70%, under silver complex catalysis). m.p. 177.5°C. IR (ATR) *ν*_max⁡_/cm^−1^: 3150, 1600, 1350, 1260. ^1^H NMR (500 MHz, CDCl_3_) *δ*/ppm: 3.31 (s, 4H), 3.93 (s, 4H), 4.60 (s, 4H), 7.82 (m, 8H), 7.77 (m, 2H), and 8.25 (s, 2H). ^13^C NMR (125 MHz, CDCl_3_) *δ*/ppm: 47.9 (2 × CH_2_), 67.0 (2 × CH_2_), 119.5 (2 × CH), 123.4 (4 × CH), 125.8 (2 × CH), 126.8 (4 × CH), 128.9 (2 × C), and 144.8 (2 × C). MS (EI^+^) *m/z* (%): 418 (23), 324 (64), 288 (100), 290 (80), 254 (70), and 161 (54). HRMS (EI): calcd. for C_20_H_20_N_6_O calcd. 360.1699, found 360.1701.

### 2.13.  4-Chlorophenoxymethyl -1-{2-[2-(4-chlorophenoxymethyl-1,2,3-triazol-1-yl)-ethoxy]-ethyl}-1,2,3-triazole

1-Chloro-4-prop-2-ynyloxy-benzene and 1-azido-2-(2-azido-ethoxy)-ethane afforded 4-chlorophenoxymethyl-1-{2-[2-(4-chlorophenoxymethyl-1,2,3-triazol-1-yl)-ethoxy]-ethyl}-1,2,3-triazole as white solid. Yields: 357.2 mg (73%, under AgCl catalysis) and 355.9 mg (71%, under silver complex catalysis). m.p. 185°C. IR (ATR) *ν*_max⁡_/cm^−1^: 3150, 1600, 1350, 1260. ^1^H NMR (500 MHz, CDCl_3_) *δ*/ppm: 3.31 (s, 4H), 3.93 (s, 4H), 4.60 (s, 4H), 7.82 (m, 8H), 7.77 (m, 2H), and 8.25 (s, 2H). ^13^C NMR (125 MHz, CDCl_3_) *δ*/ppm: 49.6 (2 × CH_2_), 61.3 (2 × CH_2_), 68.6 (2 × CH_2_), 120.9 (4 × CH), 123.6 (2 × CH), 124.3 (2 × C), 129.9 (4 × CH), 142.7 (2 × C), and 158.4 (2 × C). MS (EI^+^) *m/z* (%): 488 (20), 266 (100). HRMS (EI): calcd. for C_22_H_22_Cl_2_N_6_O_3_ calcd. 488.1130, found 488.1132.

## 3. Results and Discussion

**Scheme 1:**
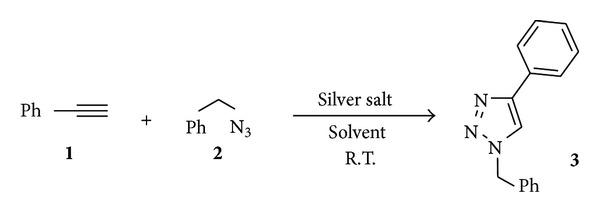
Cycloaddition between azide **2** and alkyne **1** using silver salts.

Our initial studies were carried out on phenylacetylene (**1**) and benzyl azide (**2**) using catalytic amounts of diverse silver salts (Scheme 1). According to McNulty and coworkers [9, 10], only starting materials were detected using silver nitrate as catalyst, and a similar result was obtained with silver sulfate. However, in presence of catalytic amounts of silver chloride, alkyne **1** and azide **2** afforded 1-benzyl-4-phenyl-1,2,3-triazole (**3**) as only reaction product.

This last result motivated us to investigate the role of the solvent and catalyst ratio in this process. The results, summarized in Table 1, indicate that a mixture 3 : 1 acetone-H_2_O affords the best yields compared to other polar and unpolar solvents. In addition, we carried out reactions between alkyne **1** and azide **2** with different catalyst concentrations. Although silver chloride is effective as catalyst in 1% mol concentration, the best results were obtained using 3.5% mol catalyst, affording triazole **3** in highest yield (64%) and shortest time (24 h, Table 1).

In order to explore the reaction scope, diverse alkynes and azides were reacted in presence of catalytic silver chloride. In all cases, 1,2,3-triazoles were obtained in moderate to good yields (Table 2). As conventional CuAAC, this process does not require additional special conditions, such as inert atmospheres; moreover, the workup and purification of final products are similar to those described for copper catalyzed reactions.

Another highlight of this procedure is that it does not necessitate additional reagents such as bases or reducing agents which are essential for CuAAC in some cases [2]. Other oxidation products like bistriazoles which are observed in some CuAAC reactions [19] were not detected in this process (Figure 2).

On the other hand, *N*-heterocyclic carbene metal complexes have emerged in recent years as a useful source of catalysts with many applications in a wide variety of processes [20–25]. In this context, “abnormal” *N*-heterocyclic carbene (aNHC) metal complexes represent the newest study trend in this area [26–28]. In these metal complexes, the carbene carbon in imidazolylidene ring is located at C4/C5 which is coordinated to a transition metal. This bonding, together with the reduced heteroatom stabilization, the lack of delocalization through the heterocycle, and the high steric hindrance, provides a unique electronic behaviour which develops aNHCs into interesting ligands for catalysis.

**Scheme 2:**
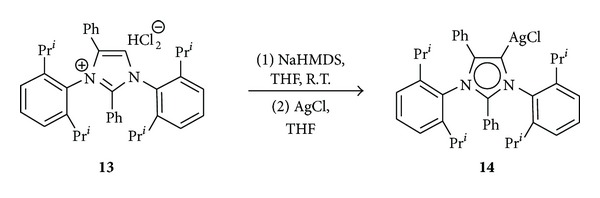
Preparation of carbene complex **14** from imidazolium salt **13**.

Inspired by these facts, we decided to design a novel catalyst for AAC from a novel kind of stable aNHCs that have been described previously by Aldeco-Perez et al. [11]. Thus, 1,3-bis-(2,6-diisopropylphenyl)-2,4-diphenylimidazolium chloride **13** was successively treated with two equivalents NaHMDS and AgCl at room temperature to afford the silver complex **14** as a light gray powder in 54% yield (Scheme 2).

**Figure 1:**
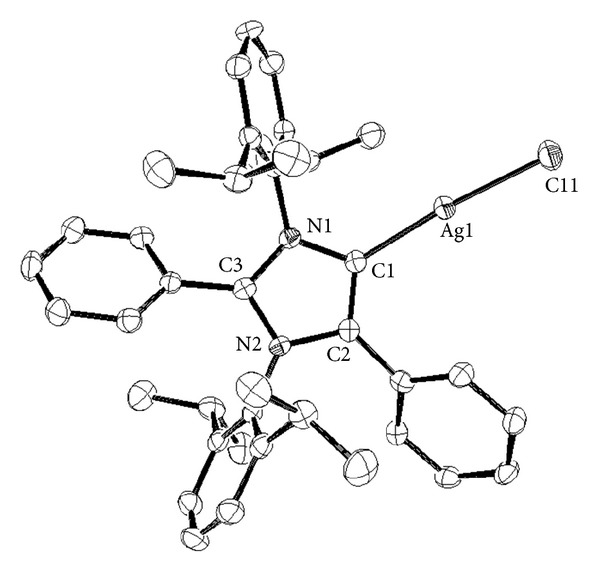
ORTEP representations for silver carbene complex **14**.

**Figure 2:**
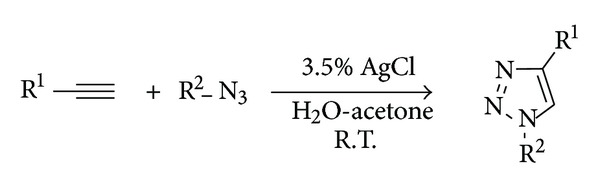


The silver carbene complex **14** was characterized by the conventional spectroscopic techniques. In addition, complex **14** was a crystalline solid which was studied by X-ray crystallography, confirming the proposed structure for this compound (Figure 1). To the best of our knowledge, this is the first example about the synthesis and isolation of a silver aNHC complex.

X-ray data reveal that silver (I) complex is a two-coordinate compound, showing an environment nearly linear (C1-Ag-Cl angle 172.9 (6) Å). Ag-C1 bond distance is 2.071 (2) Å, in agreement with other silver monocarbene complexes (derived from NHCs) reported (from 2.056 to 2.094 Å) [29]. As expected, silver-carbon bond is longer than the gold carbene bond, found for the analogous aNHC-AuCl (1.981 (2) Å) [11]. 

Ag-Cl distance is as well in the range of silver chloride complexes (2.3278 (6) Å), and no evident argentophilic interactions were detected.

Preliminary experiments demonstrated that complex **14** is an efficient catalyst for synthesis of triazole **3** from AAC in several solvents at room temperature, affording the best yields with THF (Table 3). A noteworthy feature is that complex **14** exhibits a remarkable catalytic activity in this process, requiring only 0.5% mol catalyst; the reaction also proceeds with 0.1% mol catalyst, but with a lower yield. Thus, the change of ligand increases the catalytic ability in the silver complex, similar to some ligands based on *N*-heterocyclic carbenes [30–32], phosphines [33, 34], phosphoramidites [35], or phosphinites [36] which stabilize and improve the catalytic power in some copper complexes.

**Figure 3:**
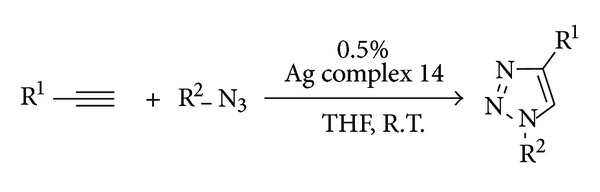


Furthermore, complex **14** displayed a broad functional group tolerance when various alkynes and azides were treated with catalytic complex **14** to give the corresponding 1,2,3-triazoles with moderate-good yields (Table 4). In consequence, complex **14** resulted in an effective catalyst for AAC (Figure 3).

Although the mechanistic details are not clear, previous experiments by McNulty and coworkers [9, 10] showed that silver acetylide formation plays an important role in the catalytic cycle as well as other intermediates [37].

**Scheme 3:**
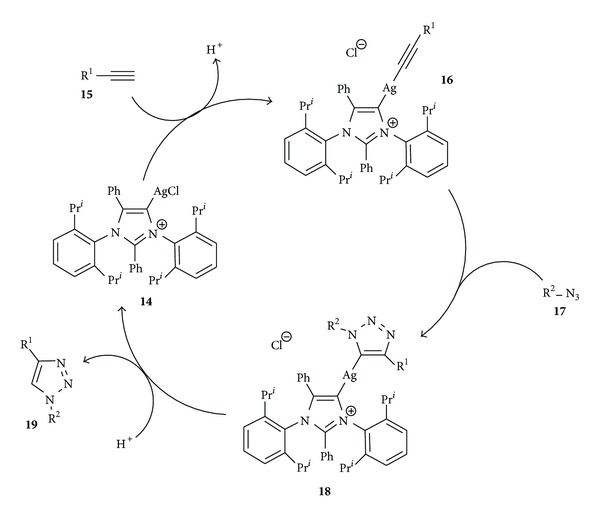
A plausible reaction mechanism of catalytic cycle.

A rational explanation of the high catalytic activity of complex **14** in AAC should include the above mentioned elements together with the ability of the aNHC ligand to protect the silver (I) ion and stabilize some intermediates such as silver acetylides. Thus, the reaction mechanism probably occurs through the formation of an intermediate like the silver acetylide complex **16** derived from an alkyne **15** and silver complex **14**, with the subsequent hydrogen interchange from the alkyne carbon to the imidazolylidene ring (Scheme 3). In the next step, an azide **17** is incorporated to acetylide complex **16** to form a silver triazolide **18**, similar to the formation of some copper triazolides [38]. Finally, a second hydrogen transfer might take place to afford the corresponding triazole **19** and regenerating the catalyst **14**.

## 4. Conclusions

In conclusion, silver compounds represent a new source of potential catalysts for AAC. In this case, silver chloride by itself can easily catalyze the cycloaddition of diverse alkynes and azides in good yields. The catalytic activity in this kind of compounds is improved with the introduction of aNHC ligands avoiding side reactions and facilitating the purification of final products, such as many copper catalyzed cycloadditions. These last results display alternative synthesis methodologies to obtain diverse 1,2,3-triazoles, which complement and extend the results of McNulty and coworkers [9, 10]. The simplicity of the method suggests that this route to 1,2,3-triazoles will enjoy widespread application.

## Figures and Tables

**Table 1:** Synthesis of triazole **3** catalyzed by silver salts.

Entry	Silver salt	Catalyst ratio (% mol)	Solvent	Reaction time (h)	% Yield
1	AgNO_3_	1	H_2_O	48	0
2	AgNO_3_	1	THF	48	8
3	Ag_2_SO_4_	1	H_2_O	48	0
4	Ag_2_SO_4_	1	THF	48	0
5	Ag_2_O	1	H_2_O	48	0
6	Ag_2_O	1	THF	48	0
7	AgCl	0.5	Acetone	24	35
8	AgCl	0.5	THF	24	27
9	AgCl	0.6	H_2_O	24	35
10	AgCl	0.6	Acetone	24	47
11	AgCl	0.6	H_2_O/acetone	24	55
12	AgCl	0.6	CH_2_Cl_2_	24	0
13	AgCl	0.6	CH_3_OH	24	31
14	AgCl	0.6	THF	24	25
15	AgCl	1	H_2_O/acetone	24	58
16	AgCl	3.5	H_2_O/acetone	24	64
17	AgCl	5	H_2_O/acetone	24	64

**Table 2:** Synthesis of triazoles catalyzed by silver chloride.

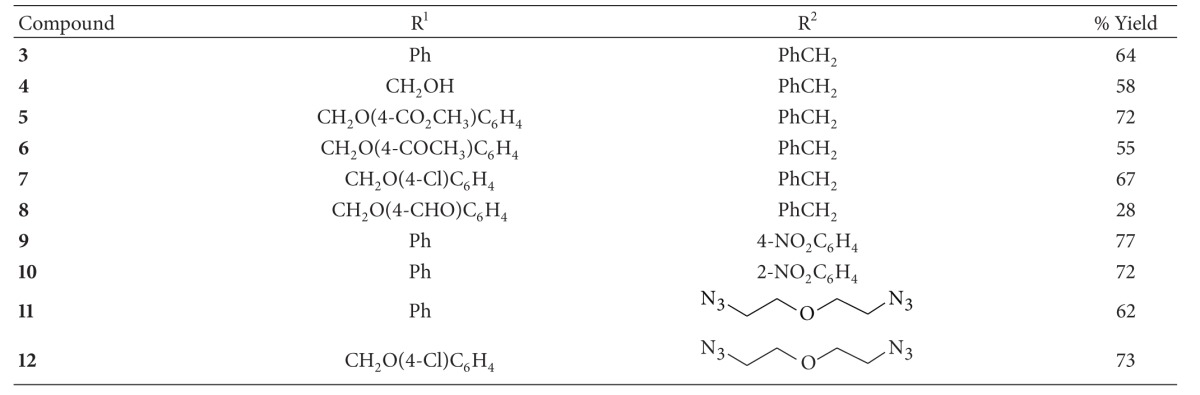

**Table 3:** Synthesis of triazole **3** catalyzed by silver complex **14**.

Entry	Catalyst ratio (% mol)	Solvent	Reaction time (h)	% Yield
1	0.6	CH_2_Cl_2_	24	58
2	0.5	CH_3_OH	24	45
3	0.5	H_2_O	24	0
4	0.5	H_2_O/acetone	15	65
5	2	THF	15	76
6	1	THF	15	75
7	0.5	THF	15	77
8	0.1	THF	15	68

**Table 4:** Synthesis of triazoles catalyzed by silver complex **14**.